# Clinical characteristics, long-term outcomes, and prognostic factors in linear IgA bullous dermatosis: A retrospective cohort study across the age spectrum in China, 2008–2025

**DOI:** 10.1016/j.jdin.2026.09.002

**Published:** 2026-09-21

**Authors:** Zhe Wang, Zhenzhen Wang, Wenchao Li, Anqi Cheng, Jianwei Li, Xiaojiao Tu, Xitong Zhao, Baoqi Yang, Yonghu Sun, Qing Yang, Furen Zhang, Hong Liu

**Affiliations:** a Dermatology Hospital of Shandong First Medical University, Jinan, Shandong, China; b Shandong Provincial Institute of Dermatology and Venereology, Shandong Academy of Medical Sciences, Jinan, Shandong, China

**Keywords:** clinical features, complete remission off therapy, epidemiology, linear IgA bullous dermatosis, long-term outcomes, relapse

*To the Editor:* Linear immunoglobulin A bullous dermatosis (LABD) is a rare autoimmune subepidermal blistering disease.^1^^,^^2^ Its long-term clinical outcomes remain poorly characterized, and complete remission off therapy (CROT) has rarely been specifically assessed, particularly across the full age spectrum, leading to a fragmented understanding of the disease.^1^, ^2^, ^3^ We therefore evaluated CROT, relapse, and their associated factors, and compared pediatric- and adult-onset LABD.

We retrospectively reviewed patients with LABD treated at our center from 2008 to 2025 (Supplementary Fig 1, available via Mendeley at https://data.mendeley.com/datasets/8sjkhbjw6x/1). The study was approved by our institutional ethics committee (approval no. 20260117KYKTKS001). Patients were classified as pediatric-onset (<18 years) or adult-onset. CROT was defined as complete clearance for ≥2 months off therapy, and complete remission during therapy as complete clearance during treatment. Relapse was defined as >3 new lesions within 1 month not healing within 1 week or extension of existing lesions after disease control.^4^ Relapse analyses were restricted to 66 patients who had achieved disease control before relapse assessment, regardless of their remission status at final follow-up. Factors associated with CROT and relapses were evaluated using age-group-adjusted Cox and logistic regression models, respectively, with additional age-stratified analyses (Supplementary Tables IV and V, available via Mendeley at https://data.mendeley.com/datasets/8sjkhbjw6x/1). All supplementary materials, available via Mendeley at https://data.mendeley.com/datasets/8sjkhbjw6x/1.

Among 71 patients (24 pediatric-onset and 47 adult-onset), the median follow-up was 47 months. At final follow-up, 63 patients (88.7%) were in clinical remission, including 30 (42.3%) in CROT and 33 (46.5%) in complete remission during therapy. The cumulative CROT rates at 1, 3, and 5 years were 17.4%, 34.9%, and 50.1%, respectively (Supplementary Fig 2, available via Mendeley at https://data.mendeley.com/datasets/8sjkhbjw6x/1). Pediatric-onset patients had higher corresponding rates than adults (32.3% vs 9.4%, 44.6% vs 28.8%, and 58.5% vs 44.0%), with a significant difference at 1 year (*P* = .040) (Fig 1; Table I). Relapse occurred in 39 of 66 eligible patients (59.1%) and was comparable between pediatric- and adult-onset patients (50.0% vs 64.3%, *P* = .304); 71.8% of relapses occurred following treatment reduction or discontinuation (Table I).

**Fig 1:**
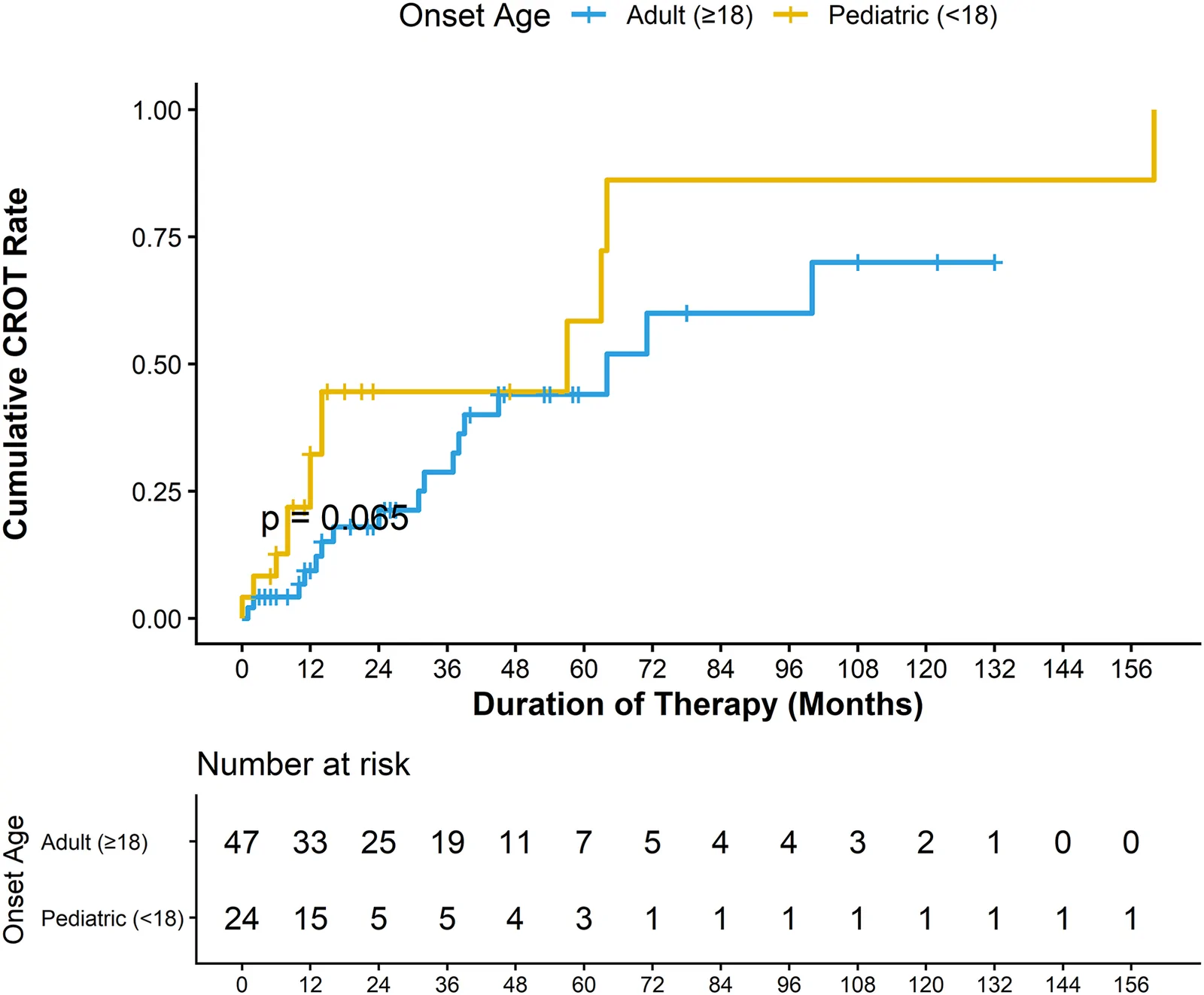
Linear IgA bullous dermatosis. Kaplan–Meier survival curves comparing the estimated cumulative complete remission off therapy (CROT) rates between pediatric-onset and adult-onset patients. *IgA*, Immunoglobulin A.

**Table I:** Key clinical characteristics, long-term outcomes, and prognostic factors

Key clinical characteristics and long-term outcomes				
Characteristic	Patients (*n* = 71)	Pediatric (*n* = 24)	Adults (*n* = 47)	*P*-value
Age at onset, y	33 (9, 56)	6.5 (5.0, 9.2)	44.0 (34.5, 67.0)	NA
Follow-up time, mo	47 (21, 83)	33.5 (14.3, 64.5)	56.0 (26.0, 85.5)	.206
IIF (+), *n*/*N* (%)	24/59 (40.7%)	13/20 (65.0%)	11/39 (28.2%)	.011
HLA-DQB1∗02:01 (+), *n*/*N* (%)	18/65 (27.7%)	11/22 (50.0%)	7/43 (16.3%)	.007
Baseline comorbidities, *n*/*N* (%)	33/71 (46.5%)	3/24 (12.5%)	30/47 (63.8%)	<.001
Pruritus NRS at diagnosis	7 (5, 9)	6 (4, 8)	8 (5, 10)	.044
DLQI at diagnosis	8 (4, 12)	4 (2, 6)	10 (7, 13)	<.001
Clinical remission, *n*/*N* (%)	63/71 (88.7%)	24/24 (100.0%)	39/47 (83.0%)	.045
CROT at final follow-up, *n*/*N* (%)	30/71 (42.3%)	13/24 (54.2%)	17/47 (36.2%)	NA
1-y cumulative CROT rate	17.4%	32.3%	9.4%	.040
3-y cumulative CROT rate	34.9%	44.6%	28.8%	.257
5-y cumulative CROT rate	50.1%	58.5%	44.0%	.407
Relapse among eligible patients, *n*/*N* (%)	39/66 (59.1%)	12/24 (50.0%)	27/42 (64.3%)	.304
Relapse following tapering/discontinuation, *n*/*N* (%)	28/39 (71.8%)	11/12 (91.7%)	17/27 (63.0%)	.122

Age-group-adjusted prognostic factors			
Outcome	Factor	Adjusted estimate (95% CI)	*P*-value
CROT	Baseline comorbidities	HR, 0.33 (0.13-0.83)	.019
Relapse	Pruritus NRS at diagnosis	OR, 1.27 (1.03-1.56)	.024

Continuous variables are presented as median (interquartile range), and categorical variables as *n*/*N* (%). Cumulative CROT rates were estimated using Kaplan–Meier analysis. Adjusted estimates were obtained from separate Cox proportional hazards and logistic regression models adjusted for age group.

Relapse analyses were restricted to 66 patients (24 pediatric-onset and 42 adult-onset) who had achieved disease control before relapse assessment.

*CI*, Confidence interval; *CROT*, complete remission off therapy; *DLQI*, Dermatology Life Quality Index; *HLA*, human leukocyte antigen; *HR*, hazard ratio; *IIF*, indirect immunofluorescence; *LABD*, linear IgA bullous dermatosis; *NA*, not applicable; *NRS*, Numerical Rating Scale; *OR*, odds ratio.

Pediatric-onset patients had higher indirect immunofluorescence positivity (65.0% vs 28.2%, *P* = .011) and human leukocyte antigen-DQB1∗02:01 (major histocompatibility complex, class II, DQ beta 1) carriage (50.0% vs 16.3%, *P* = .007),^5^ but lower baseline comorbidity burden (12.5% vs 63.8%, *P* < .001), pruritus, and Dermatology Life Quality Index scores than adults (Table I; Supplementary Table I, available via Mendeley at https://data.mendeley.com/datasets/8sjkhbjw6x/1). In age-group-adjusted models, baseline comorbidities were associated with reduced CROT (hazard ratio, 0.33; 95% CI, 0.13–0.83; *P* = .019), whereas higher baseline pruritus Numerical Rating Scale was associated with relapse (OR, 1.27; 95% CI, 1.03–1.56; *P* = .024) (Table I; Supplementary Tables II and III, available via Mendeley at https://data.mendeley.com/datasets/8sjkhbjw6x/1). Age-stratified analyses showed that higher baseline Pruritus Numerical Rating Scale was associated with relapse in adults (OR = 1.32, *P* = .04), while a similar direction was observed in pediatric patients (OR = 1.18, *P* = .33), although the pediatric analysis was limited by sample size (Supplementary Table V, available via Mendeley at https://data.mendeley.com/datasets/8sjkhbjw6x/1).

Although most patients were in clinical remission at final follow-up, fewer than half achieved CROT, and relapse was common across both age groups. Pediatric- and adult-onset LABD differed in clinical and prognostic profiles, supporting individualized long-term follow-up and cautious treatment tapering.

### Declaration of generative AI and AI-assisted technologies in the writing process

During the preparation of this work, the authors used Gemini for manuscript organization, English-language polishing, and formatting. After using this tool, the authors reviewed and edited the content as needed and take full responsibility for the content of the article.

## Conflicts of interest

None disclosed.
